# Robotic versus Electromagnetic Bronchoscopy for Peripheral Pulmonary Lesions: A Randomized Trial (RELIANT)

**DOI:** 10.1164/rccm.202409-1846OC

**Published:** 2025-06-03

**Authors:** Rafael Paez, Robert J. Lentz, Jennifer D. Duke, Justin K. Siemann, Cristina Salmon, Greta J. Dahlberg, Ankush P. Ratwani, Jonathan D. Casey, Heidi Chen, Sheau-Chiann Chen, Samira Shojaee, Otis B. Rickman, Cheryl L. Gatto, Todd W. Rice, Fabien Maldonado

**Affiliations:** ^1^ Department of Medicine, Division of Allergy, Pulmonary and Critical Care Medicine,; ^2^ Vanderbilt Institute for Clinical and Translational Research,; ^3^ Department of Biostatistics, and; ^4^ Department of Emergency Medicine, Vanderbilt University Medical Center, Nashville, Tennessee;; ^5^ Department of Medicine, Division of Pulmonary, Allergy and Critical Care Medicine, Duke University, Durham, North Carolina; and; ^6^ University of Tennessee Health Science Center, Ascension Saint Thomas, Nashville, Tennessee

**Keywords:** bronchoscopy, electromagnetic navigational bronchoscopy, robotic-assisted bronchoscopy, peripheral lung lesions, diagnostic yield

## Abstract

**Rationale:**

Robotic-assisted bronchoscopy has emerged as an alternative to electromagnetic navigational bronchoscopy for patients undergoing bronchoscopic biopsy of a peripheral pulmonary lesion. Although both platforms are routinely used in clinical practice, comparative effectiveness data are lacking.

**Objectives:**

We sought to compare the effectiveness of robotic-assisted and electromagnetic navigational bronchoscopy for the evaluation of peripheral pulmonary lesions.

**Methods:**

In an investigator-initiated, single-center, cluster-randomized noninferiority trial, we assigned patients undergoing diagnostic bronchoscopy for evaluation of a peripheral pulmonary lesion to either robotic-assisted or electromagnetic navigational bronchoscopy. The cluster randomization unit was the operating room in which patients were scheduled. The primary outcome was the diagnostic yield of the procedure, defined as the proportion of cases yielding lesional tissue. Secondary and safety outcomes included procedure duration and complications.

**Measurements and Main Results:**

Among the 411 patients included in the modified intention-to-treat analysis, lesional tissue was obtained in 158 of 203 (77.8%) patients in the robotic-assisted group and 157 of 208 (75.5%) patients in the electromagnetic group; the *P* value for noninferiority was 0.007. The median duration of bronchoscopy was 37 minutes in the robotic-assisted group and 32 minutes in the electromagnetic group (difference, 5 min; 95% confidence interval = 2.0–7.7). Pneumothorax occurred in 4 patients in the robotic-assisted group and 6 patients in the electromagnetic group.

**Conclusions:**

In patients undergoing bronchoscopy for the evaluation of a peripheral pulmonary lesion, the diagnostic yield of robotic-assisted bronchoscopy was not inferior to that of electromagnetic navigation bronchoscopy.

Clinical trial registered with www.clinicaltrials.gov (NCT 05705544).

**At a Glance Commentary**

**Scientific Knowledge on the Subject**

Robotic-assisted bronchoscopy and electromagnetic navigational bronchoscopy are routinely used in clinical practice to biopsy peripheral pulmonary lesions; however, comparative effectiveness data are lacking.

**What This Study Adds to the Field**

The Robotic versus Electromagnetic Bronchoscopy for Pulmonary Lesion Assessment (or, RELIANT) trial is the first cluster-randomized trial in interventional pulmonology and may serve as an innovative paradigm for future trials evaluating medical devices. The results of the RELIANT trial suggest that, in the hands of trained operators, both robotic-assisted bronchoscopy and electromagnetic navigational bronchoscopy are reasonable approaches for the evaluation of peripheral pulmonary lesions.

Millions of patients are diagnosed with peripheral pulmonary lesions each year (1), and advanced navigational bronchoscopy is increasingly used when biopsy is needed for diagnosis (2, 3).

Navigational bronchoscopy refers to a procedure in which a steerable catheter is combined with preprocedural and intraprocedural imaging to navigate to a pulmonary lesion (3). Electromagnetic navigational bronchoscopy (ENB) uses low-frequency electromagnetic waves and a locatable guide to allow precise tracking of the position and orientation of the steerable catheter as it navigates through airways to a peripheral target. ENB with integrated digital tomosynthesis has substantially improved the diagnostic yield over prior techniques that were limited to two-dimensional fluoroscopic images (4). In recent years, robotic-assisted bronchoscopy (RAB) has emerged as a promising alternative to ENB (5–7). RAB uses shape-sensing technology to track the position of the catheter within the airways.

Both ENB and RAB were cleared for clinical use through the Food and Drug Administration 510(k) pathway, which only requires that a device appears similar in safety and effectiveness compared with existing commercially available devices (8, 9). The potential benefits of RAB include increased stability, on-demand distal catheter articulation, and a favorable learning curve. Data comparing the two devices, however, are limited to small retrospective studies, which have not shown significant differences in safety or effectiveness across the two devices (5–7).

To understand whether shape-sensing, RAB is as effective as ENB with integrated digital tomosynthesis for evaluation of peripheral pulmonary lesions, we conducted the Robotic versus Electromagnetic Bronchoscopy for Pulmonary Lesion Assessment (RELIANT) trial. We hypothesized that the diagnostic yield of RAB would be noninferior to that of ENB.

## Methods

### Study Design and Oversight

The RELIANT trial was an investigator-initiated, unblinded, cluster-randomized, noninferiority trial. The study was embedded within routine clinical care with the only aspect of clinical care controlled by the protocol being the navigation platform used in a particular operating room on a given day. A noninferiority was selected because, at the time of trial design, there were very little comparative data between these two bronchoscopic modalities. The data available at the time of study design suggested similar diagnostic yield, with a slight, albeit nonsignificant, advantage for ENB (5). RAB offers several distinct advantages over ENB, such as a more favorable learning curve, catheter stability, the ability to finely adjust the tip of the catheter for optimal alignment with the target lesion, and the possible integration with cone-beam computed tomography (CT). Because of these advantages, noninferiority to ENB in terms of diagnostic yield would offer compelling evidence for the diagnostic utility of RAB. All patients provided written informed consent for the use of their data before the procedure. The trial was approved by the Vanderbilt University Medical Center Institutional Review Board (#221,255), was registered at ClinicalTrials.gov (NCT 05705544) before initiation, and was overseen by an independent data and safety monitoring board. The protocol and statistical analysis plan, available in the Supplementary Appendix, were published before the conclusion of enrollment (10).

### Trial Setting and Patient Selection

The study was conducted in two dedicated bronchoscopy operating rooms at a single academic medical center. Patients were eligible if they were at least 18 years of age and scheduled to undergo navigational bronchoscopy for the evaluation of a peripheral pulmonary lesion. Patients were ineligible if they were enrolled in another clinical trial that required the use of ENB or if they declined to participate.

### Randomization and Blinding

Randomization occurred at the level of the operating room (cluster randomization). In the RELIANT trial, operating rooms were randomized daily to RAB or ENB. A cluster design was chosen as logistical constrains made individual-level randomization impracticable. Both RAB and ENB platforms are mobile, but setting them up requires time and resources. At our institution, bronchoscopic biopsies for peripheral pulmonary lesions are performed concurrently in two operating rooms multiple times per week, with each room dedicated to one of the two navigational platforms. These platforms are set up at the start of the day and used for every patient undergoing biopsy in that room. Once the platforms are set up, workflow constraints prevent moving the platforms between operating rooms, as one platform may be in use while another patient is ready for navigation bronchoscopy in the other room (10). Allocation was concealed in sealed, opaque envelopes and opened by the operating room staff or the proceduralist each morning on days when at least one navigational bronchoscopy case was scheduled. A biostatistician who was not involved in patient care generated the randomization sequence using randomly permuted blocks stratified by the day of the week. Proceduralists, patients, and schedulers were blinded until the day of the procedure to preclude intentional scheduling of a given case to a specific navigational platform. Bronchoscopies were scheduled days or weeks before randomization without knowledge of group assignment.

### Trial Interventions

For patients assigned to the RAB group, bronchoscopists were instructed to perform navigational bronchoscopy with RAB. The RAB platform used in the study setting was the Ion Endoluminal System (Intuitive Surgical). After an initial airway inspection and clearance using a standard flexible bronchoscope (BF-Q190, Olympus), the RAB arm was magnetically docked to the endotracheal tube, and the catheter was introduced through the coupler into the airways. Registration and navigation to the lesion of interest were performed as previously described (5). Mobile cone-beam CT (OEC 3D, GE OEC Medical Systems), not integrated with the Ion platform, was available to provide intraprocedural cross-sectional imaging for patients assigned to the RAB group at the discretion of the bronchoscopist.

For patients assigned to the ENB group, bronchoscopists were instructed to perform navigational bronchoscopy with ENB. The ENB platform used in the study setting was Illumisite (Medtronic) with integrated intraprocedural digital tomosynthesis. After an initial airway inspection and clearance, the locatable guide with the extended working channel was inserted in the therapeutic bronchoscope (BF-TH190, Olympus). Registration and navigation to the lesion of interest were performed as previously described (4). Use of integrated digital tomosynthesis was at the discretion of the bronchoscopist.

In both trial groups, patients received care dictated by clinical protocols that have been previously described (4, 5), which include: general anesthesia with neuromuscular blockade, placement of an 8.5-mm endotracheal tube, invasive mechanical ventilation with a recruitment maneuver followed by ventilation with a positive end expiratory pressure of 15 cm of water, and weaning of the fraction of inspired oxygen to the lowest value needed to maintain an oxygen saturation over 90% (to limit atelectasis during the procedure). Radial endobronchial ultrasound was used in all procedures. Tissue samples were obtained using transbronchial needle aspiration, biopsy forceps, cryoprobes, and/or other sampling techniques at the discretion of the bronchoscopist. Rapid onsite cytological evaluation was available in most cases to assess specimen adequacy. Pneumothorax was assessed in all patients with two-dimensional fluoroscopy with additional postprocedure imaging at the discretion of the proceduralist.

### Data Collection

Bronchoscopy data were collected and entered into a REDCap database (11) by the bronchoscopist immediately after the procedure. Trial personnel reviewed the medical record to collect data on patients’ baseline characteristics and clinical outcomes.

### Outcomes

The primary outcome was diagnostic yield, defined as the proportion of procedures that resulted in acquisition of lesional tissue (12). Lesional tissue was defined by the presence of pathological findings that explained the presence of a pulmonary lesion and were sufficient to inform patient care. The following specific pathological findings were considered diagnostic: malignancy and specific benign pathologic finding, including organizing pneumonia, frank purulence or robust neutrophilic inflammation, granulomatous inflammation, and other less common specific benign findings, such as hamartoma, or amyloidoma. Biopsies that did not meet any of the aforementioned pathological criteria were adjudicated as nondiagnostic, including biopsies with normal lung parenchyma, atypia not diagnostic of malignancy, and nonspecific inflammation.

All biopsy specimens were interpreted by lung pathologists who were blinded to group assignment. For biopsies that did not reveal malignancy, a panel of four pulmonologists who were blinded to group assignment adjudicated samples as diagnostic or nondiagnostic. Adjudication of a nonmalignant sample as diagnostic required consensus among all reviewers. If consensus was not achieved, the biopsy was adjudicated as nondiagnostic. If navigational bronchoscopy was performed but biopsies were not obtained (e.g., unable to localize the lesion or lesion resolved), the procedure was considered nondiagnostic.

The prespecified secondary outcome was duration of the navigation procedure, defined as time from beginning of registration to removal of the catheter after completion of navigation procedures. Safety outcomes focused on procedural complications within 7 days of the bronchoscopy, including pneumothorax, clinically significant bronchopulmonary hemorrhage, respiratory failure, and major complications of anesthesia including arrhythmia, acute coronary syndrome, stroke, and postprocedural hypotension.

### Statistical Analysis

The statistical analysis plan has been previously published (10). Assuming a diagnostic yield of ENB of 80% (4, 5), a noninferiority margin of 10%, an average cluster size of 2, and no intracluster correlation, it was calculated that 202 clusters (operating room days) with 404 patients would be required to provide 80% power to conclude noninferiority with a one-sided Type I error rate of 5%. The 10% noninferiority margin was chosen as a clinically significant difference in diagnostic yield, and exceeding this threshold would favor ENB over RAB. Given the small cluster size, sample size calculations assumed no intracluster correlation. To ensure that the study would have the power to evaluate the prespecified noninferiority margin in light of a lower than expected number of participants per cluster (1.8 participants per cluster), the number of clusters was increased to 229.

The primary analysis included all patients who underwent navigational bronchoscopy with either RAB or ENB. If the diagnosis was established with an alternative bronchoscopic technique (e.g., sampling central lymphadenopathy) before initiating navigational procedures and thereby obviating the need for navigational bronchoscopy, or if the procedure was aborted before navigational bronchoscopy (e.g., because of clinical instability or lack of vital equipment), the procedure was excluded from diagnostic yield calculations. If a patient underwent multiple navigational bronchoscopies during the study period, only the index bronchoscopy was included in the analysis.

The primary analysis of the primary outcome (diagnostic yield) was performed with a generalized linear mixed model. The model was covariate adjusted, including fixed effects for device assignment, lesion size, density, peripheral location, and bronchus sign. The *P* value for the comparison between RAB and ENB was calculated using a one-sided test for noninferiority. A prespecified superiority analysis was planned if the noninferiority endpoint was met consisting of an adjusted generalized linear mixed-effects model with the same covariates used in the primary analysis (10). No adjustment was made for multiplicity. The superiority analysis was conducted with a one-sided test at a significance level of 0.05.

Additional analyses of the primary outcome included a difference of two proportions calculated using the *Z* test (unpooled) statistic for noninferiority test in a cluster-randomized design. The difference in diagnostic yield with 90% confidence interval (CI) was reported. Sensitivity analyses included a per-protocol analysis (10).

In accordance with published guidelines (13), we examined whether prespecified baseline variables modified the effect of trial-group assignment on the primary outcome by introducing interaction terms between group assignment and the subgrouping variable into the generalized linear mixed model used for the primary outcome, one by one. Odds ratios (ORs) with 95% CIs were reported.

Analysis of the secondary and safety outcomes followed a similar approach as the primary outcome. We conducted additional analyses using Pearson’s chi-square test for binary and categorical outcomes and a Welch two-sample *t* test for continuous outcome. Differences were reported as point estimates and 95% CIs. The widths of the CIs were not adjusted for multiplicity and should not be used to infer definitive differences in treatment effects between the two trial groups. All statistical analyses were conducted in R (Version 4.4.0).

## Results

### Patients

**Figure 1.**
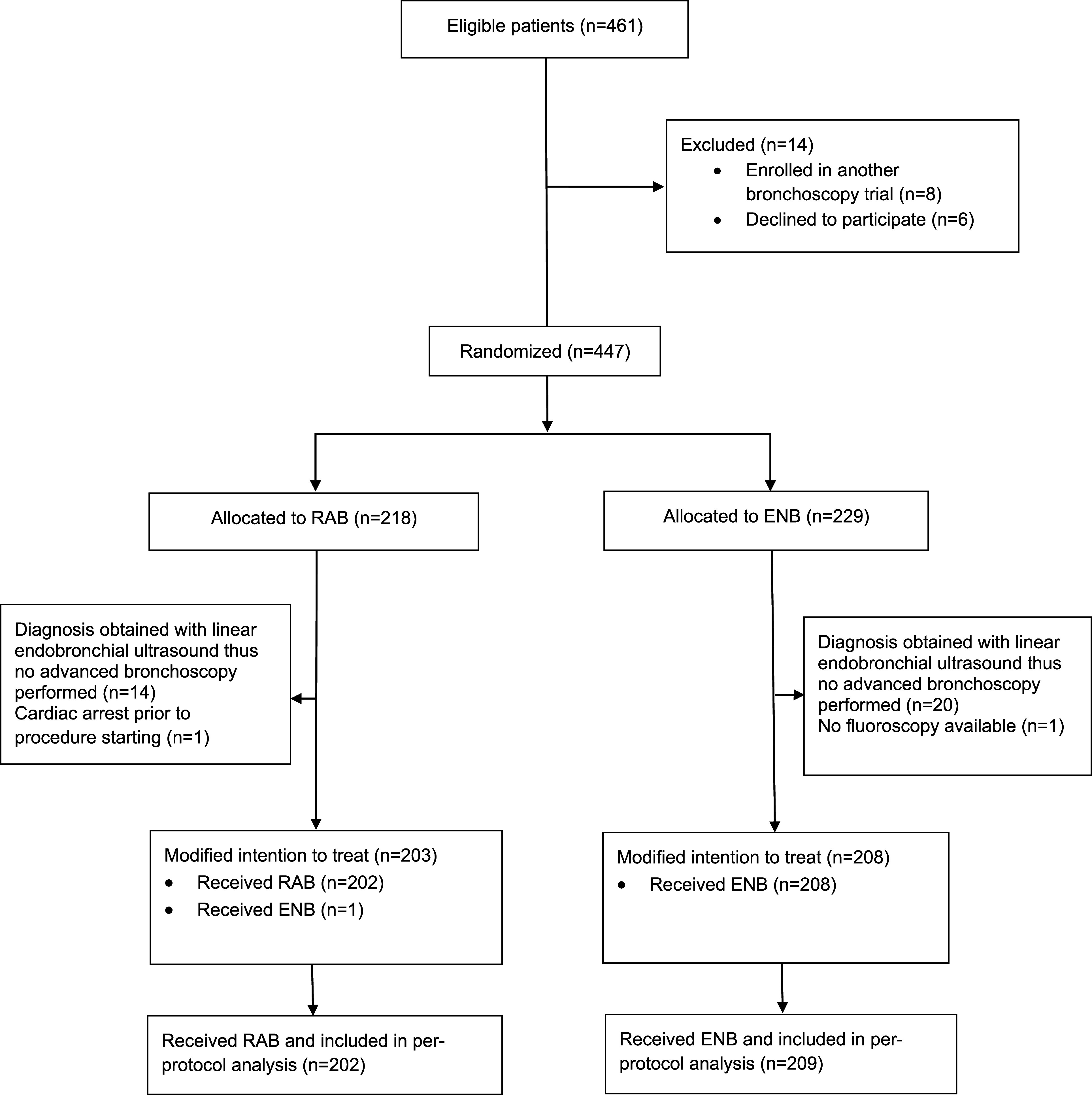
Consort Diagram. ENB = electromagnetic navigational bronchoscopy; RAB = robotic-assisted bronchoscopy.

From March 6, 2023, to April 9, 2024, 461 patients met the inclusion criteria. Of these, 447 patients met no exclusion criteria, provided informed consent, and were enrolled (Figure 1). The diagnosis was obtained with linear endobronchial ultrasound before the initiation of navigational bronchoscopy in 34 patients, and for 2 patients, the procedure was aborted before navigational bronchoscopy because of anesthetic complications (*n* = 1) and lack of fluoroscopy equipment (*n* = 1). A total of 411 patients underwent navigational bronchoscopy and were included in the modified intention-to-treat analysis. A total of 203 patients (49.4%) were assigned to the RAB group, and 208 patients (50.6%) were assigned to the ENB group. The median age of participants was 67 years old (interquartile range [IQR] = 60–74.5), and 48.9% were women. Most patients were either current (*n* = 88; 21.4%) or former smokers (*n* = 191; 46.5%) (Table 1). Concurrent malignancy was present in 22.6% (*n* = 93) of participants, and prior malignancy was present in 35.0% (*n* = 144) of participants. Most of the lesions biopsied were solid and located in the outer third of the lungs. The median lesion size was 19 mm (IQR = 13–28), and a bronchus sign (airway leading to the lesion of interest) was present in 240 cases (58.4%). The groups were well balanced regarding lesion characteristics except for spiculation, which was present in 62 patients (30.5%) in the RAB group and 41 patients (19.7%) in the ENB group.

**Table 1.** Baseline Demographic and Lesion Characteristics

Characteristic	RAB N = 203	ENB N = 208	Overall N = 411
Age in years, median [IQR]	69.0 [61.0–74.0]	67.0 [59.0–75.0]	67.0 [60.0–74.5]
Sex			
Women	101 (49.8%)	100 (48.1%)	201 (48.9%)
Men	102 (50.2%)	108 (51.9%)	210 (51.1%)
Race			
White	188 (92.6%)	189 (90.9%)	377 (91.7%)
Black or African American	15 (7.4%)	15 (7.2%)	30 (7.3%)
Asian	0 (0.0%)	3 (1.4%)	3 (0.7%)
Unknown or unable to report	0 (0.0%)	1 (0.5%)	1 (0.2%)
Ethnicity			
Non-Hispanic	198 (97.5%)	202 (97.1%)	400 (97.3%)
Hispanic	5 (2.5%)	6 (2.9%)	11 (2.7%)
Current malignancy	51 (25.1%)	42 (20.2%)	93 (22.6%)
Prior malignancy	71 (35.0%)	73 (35.1%)	144 (35.0%)
Body Mass Index, median [IQR]	26.5 [23.0–30.6]	26.8 [23.9–30.4]	26.6 [23.4–30.5]
Anticoagulation or antiplatelet	86 (42.4%)	85 (40.9%)	171 (41.6%)
Smoking history			
Current	44 (21.7%)	44 (21.2%)	88 (21.4%)
Former	97 (47.8%)	94 (45.2%)	191 (46.5%)
Never	62 (30.5%)	70 (33.7%)	132 (32.1%)
Lesion size, largest diameter (mm), median [IQR]	18.0 [12.0–28.0]	20.0 [14.0–29.0]	19.0 [13.0–28.0]
Lesion location (outer 1/3 of the chest)	139 (68.5%)	141 (67.8%)	280 (68.1%)
Lesion density (solid)	171 (84.2%)	176 (84.6%)	347 (84.4%)
Lesion spiculation	62 (30.5%)	41 (19.7%)	103 (25.1%)
Bronchus sign present	112 (55.2%)	128 (61.5%)	240 (58.4%)
Lesion distance from pleura (mm), median [IQR]	6.0 [0.0–18.0]	4.0 [0.0–14.0]	5.0 [0.0–15.5]

*Definition of abbreviations:* ENB = electromagnetic navigational bronchoscopy; RAB = robotic-assisted bronchoscopy.

### Bronchoscopy

A total of 202 of the 203 patients (99.5%) in the RAB group underwent RAB, and all 208 patients in the ENB group underwent ENB. Intraprocedural cone-beam CT was used for 110 of the 203 patients (54.2%) in the RAB group, and digital tomosynthesis was performed for 139 of the 203 patients (66.8%) in the ENB group. Radial endobronchial ultrasound was used in all cases. Transbronchial needle aspiration was the most common sampling technique and was used in 201 patients (99.0%) in the RAB group and 203 patients (97.6%) in the ENB group. Transbronchial biopsy using the 1.1-mm cryobiopsy probe (Erbe) was used in 78 patients (38.4%) in the RAB group and 76 patients (36.5%) in the ENB group. Transbronchial biopsy using forceps was used in 52 patients (25.6%) in the RAB group and 69 patients (33.2%) in the ENB group. Rapid onsite evaluation was performed in 195 of 203 (96.0%) patients in the RAB group and 198 of 208 (95.2%) patients in the ENB group (*see* Table E1 in the online supplement).

### Primary Outcome

**Figure 2.**
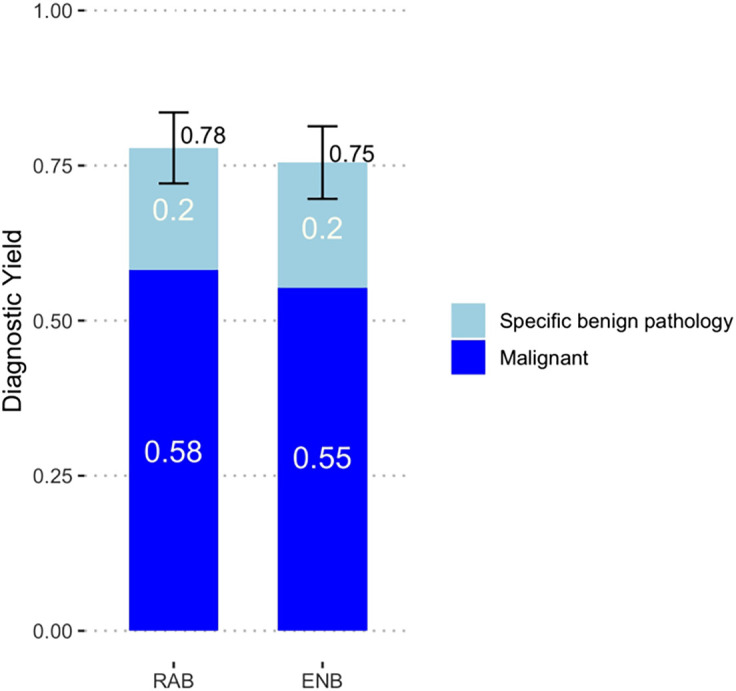
Diagnostic yield by platform. ENB = electromagnetic navigational bronchoscopy; RAB = robotic-assisted bronchoscopy.

Lesional tissue was obtained in 158 of 203 patients in the RAB group (77.8%), and 157 of 208 patients in the ENB group (75.5%) (OR, 1.18; lower boundary of 90% CI, 0.75; *P* value for noninferiority = 0.007) with a difference in diagnostic yield between the two groups of 2.3 percentage points (*Z* test statistic: 90% CI, −0.05 to 0.09; *P* value for noninferiority = 0.002) (Figure 2; Table 2; *see* Figure E1. There was no significant difference between the groups in the superiority analysis (OR, 1.23; 95% CI, 0.73 to 2.07. A diagnosis of malignancy was established in 118 patients in the RAB group (58.1%) and 115 subjects in the ENB group (55.3%). Specific benign pathology was identified in 40 subjects in the RAB group (19.7%) and 42 subjects in the ENB group (20.2%) (Table 2; *see* Table E2). Results were similar in the per-protocol analysis (*see* Figure E2). We conducted a sensitivity analysis that considered four cases of robust neutrophilic inflammation where no pathogen was isolated or where pathology did not reveal a specific finding as nondiagnostic. The diagnostic yield of RAB was 76.4%, and the diagnostic yield of ENB was 75.0%. The noninferiority findings remained unchanged; *P* value for noninferiority = 0.01.

**Table 2.** Study Outcomes and Procedure Complications

Characteristic	RAB N = 203	ENB N = 208	Overall N = 411	Difference* (95% CI)
Diagnostic yield				
Diagnostic	158 (77.8%)	157 (75.5%)	315 (76.6%)	2.4% (−6.3%, 11%)
Biopsy findings				
Malignant	118 (58.1%)	115 (55.3%)	233 (56.7%)	2.8% (−7.2%, 13%)
Specific benign pathology	40 (19.7%)	42 (20.2%)	82 (20.0%)	−0.5% (−8.7%,7.7%)
Non-diagnostic	45 (22.2%)	51 (24.5%)	96 (23.4%)	−2.4% (−11%, 6.3%)
Duration of procedure (mins), median, IQR	37.0 [29.0–48.0]	32.0 [25.0–43.3]	35.0 [27.0–45.0]	4.8 (2.0, 7.7)
Procedure complications	5 (2.5%)	12 (5.8%)	17 (4.1%)	−3.3% (−7.6%, 1.0%)
Pneumothorax	4	6	10	
Hypoxia	0	1	1	
COPD exacerbation	0	1	1	
Persistent dyspnea post-procedure	0	2	2	
Persistent cough	0	1	1	
Anesthesia complication	1	1	2	

*Definition of abbreviations:* ENB = electromagnetic navigational bronchoscopy; RAB = robotic-assisted bronchoscopy.

* Difference was calculated using Pearson’s Chi-squared test for binary/categorical outcome and Welch two sample t-test for continuous outcome.

**Figure 3.**
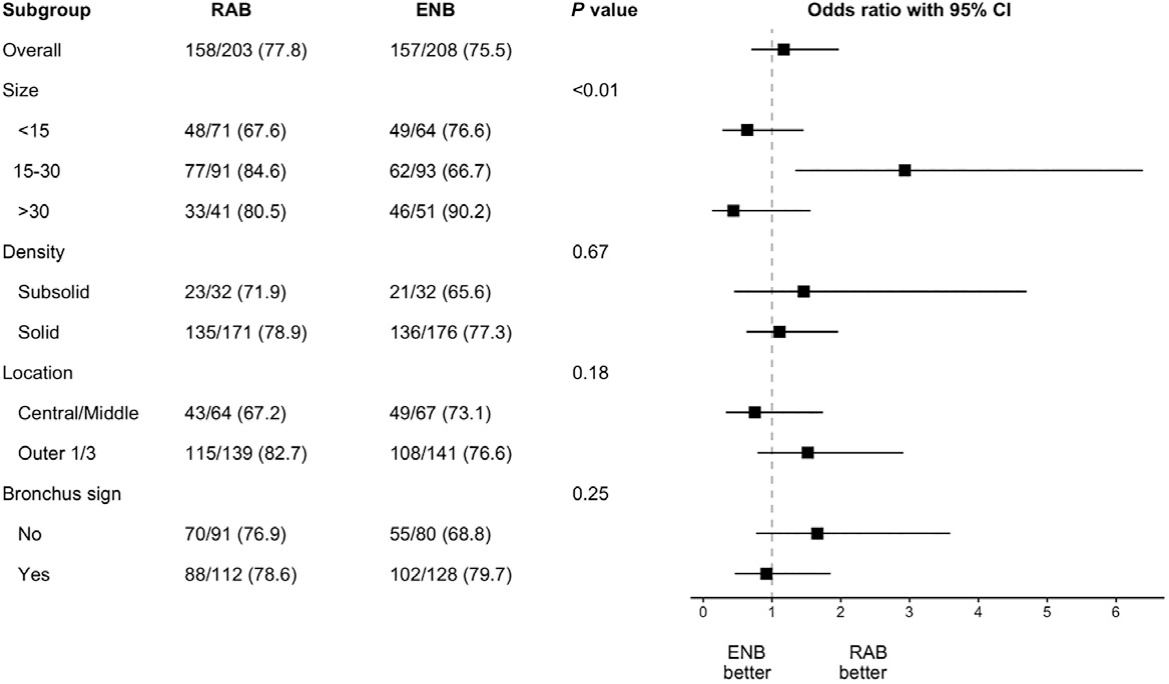
Odds ratio with 95% confidence interval for the primary outcome, diagnostic yield, in prespecified subgroups based on the generalized mixed-effect model. *P* value was used for evaluating interaction terms between platform and subgrouping variable. ENB = electromagnetic navigational bronchoscopy; RAB = robotic-assisted bronchoscopy.

Figure 3 shows the results of the primary outcome in the prespecified subgroups. The size of the nodule appeared to modify the relationship between group assignment and diagnostic yield, with effect estimates favoring RAB in nodules of intermediate size (*P* value for the interaction term between platform and nodule size = 0.007; subgroup of nodule size between 15 and 30 mm: OR, 2.93 [95% CI, 1.34 to 6.39]). None of the other characteristics appeared to modify the effect of RAB versus ENB on diagnostic yield.

Of the 110 procedures (out of 203 RAB cases) where cone-beam CT was used, 85 were diagnostic, yielding a success rate of 77%. Similarly, of the 139 procedures (out of 208 ENB cases) where digital tomosynthesis was used, 99 were diagnostic, corresponding to a success rate of 71%. It is important to note that the decision to use intraprocedural imaging was driven by data acquired during the procedure, such as nondiagnostic material on rapid onsite evaluation and/or the absence of a radial endobronchial ultrasound signature. Therefore, the impact of intraprocedural imaging cannot be definitively inferred from this data.

### Secondary, Exploratory, and Safety Outcomes

The median duration of the procedure was 37 minutes (IQR = 29–48) in the RAB group versus 32 minutes (IQR = 25–43) in the ENB group (difference, 4.8 min; 95% CI = 2.0 to 7.7; OR, 4.31; 95% CI = 1.12 to 7.51). A total of 17 complications were observed during the study: 5 (2.5%) in the RAB group and 12 (5.8%) in the ENB group (difference, −3.3% [95% CI, −7.6 to 1.0]; OR, 0.39 [95% CI, 0.12 to 1.25]) (Table 2). The most common complication was pneumothorax, which occurred in 4 patients in the RAB group and 6 patients in the ENB group.

## Discussion

Among patients undergoing bronchoscopy for evaluation of a peripheral pulmonary lesion, the diagnostic yield of RAB was not inferior to that of ENB. Additionally, there was no significant difference in the rate of complications. Although the median duration of the procedure was approximately 5 minutes longer in the RAB group, the clinical significance of this difference is unclear.

To the best of our knowledge, this is the first randomized controlled trial comparing the diagnostic yield of RAB to that of ENB. The finding that RAB is noninferior to ENB, but not significantly better, is consistent with the results of a prior retrospective study from our center (5) but differs from other studies that suggest a benefit from RAB (14). The results of the RELIANT trial suggest that, in the hands of trained operators, both RAB and ENB are reasonable approaches for evaluation of peripheral pulmonary lesions. Prior studies suggesting a benefit from RAB have largely compared patients across studies or settings in which all of the patients at a specific site received the same device. Such comparisons may be biased by differences in patient populations, differential use of cointerventions (e.g., cone-beam CT, intraprocedural digital tomosynthesis, and rapid on-site cytological evaluation), differences in proceduralist experience, and differences in the definition of diagnostic yield used to adjudicate the primary outcome (14). The results of the RELIANT trial are, therefore, timely and important.

New devices, including the bronchoscopy platforms studied in the RELIANT trial, are commonly cleared through the Food and Drug Administration 510k pathway, which requires no evidence of improved patient outcomes before commercialization, provided that safety and effectiveness appear comparable with those of existing commercially available devices (8, 9). The design of the RELIANT trial has the potential to overcome the challenge of generating clinically useful data in a rapidly evolving technological landscape. This is especially important, as devices are usually cleared for clinical use without comparative data. The primary outcome of diagnostic yield, which could be adjudicated shortly after the procedure, allowed the trial to progress from enrollment to data analysis and manuscript submission in a short period of time. This approach to trial design may allow future trials to keep up with technological innovation and rapidly generate real-world data to inform optimal patient care.

Our trial has several strengths. The trial design included randomization, concealment of the trial-group assignment until the morning of the procedure to prevent selection bias, broad eligibility criteria, and a high enrollment rate increasing the generalizability of the results. The trial had excellent adherence to group assignment. Outcome assessors were blinded to group allocation to minimize observer bias. The design of the study, which embedded trial procedures in routine clinical workflows, minimized clinician burden and allowed for efficient accrual as the study enrolled 447 participants in approximately 12 months. Although cluster-randomized trials typically have less statistical power than trials with individual patient randomization, the small cluster size in the RELIANT trial (1.8 patients per cluster) approximated individual patient randomization.

Our study also has several limitations. As a single-center trial conducted among experienced operators, the results may not generalize to all settings. However, this also mitigated potential confounding related to differential experience across the two platforms. Knowledge of group assignment may be a source of bias in some cluster-randomized trials but, in this study, allocation was concealed until patients were assigned to their cluster, minimizing opportunities for selection bias during scheduling or conduct of the procedure. Given the nature of the intervention, blinding was infeasible after enrollment, which could introduce operational bias related to differential care by group, but a careful reporting of cointerventions did not suggest any differences between groups. Finally, it is possible that future technological improvements could affect the relative effectiveness of RAB and ENB, necessitating the need for additional research. Although the cone-beam CT scan was available and routinely used, it was not integrated with the RAB platform, and correction for CT-to-body divergence was performed manually after review of multiplanar reconstructions by the clinician. It remains unclear whether integration, which allows automated correction for CT-to-body divergence, will increase the diagnostic yield of RAB. Integration was available for digital tomosynthesis during ENB procedures (15, 16).

In summary, for patients undergoing bronchoscopy for the evaluation of a peripheral pulmonary lesion, the diagnostic yield of RAB is not inferior to that of ENB. Both devices have similar safety profiles. As the first cluster-randomized multiple-crossover trial in interventional pulmonology, the RELIANT trial serves as an innovative paradigm for future trials evaluating new medical devices.
